# Lewy body diseases and the gut

**DOI:** 10.1186/s13024-025-00804-5

**Published:** 2025-01-30

**Authors:** Timothy R. Sampson, Malú Gámez Tansey, Andrew B. West, Rodger A. Liddle

**Affiliations:** 1 Department of Cell Biology, Emory University School of Medicine, Atlanta, GA 30329 USA; 2 Aligning Science Across Parkinson’s (ASAP) Collaborative Research Network, Chevy Chase, MD 20815 USA; 3 Department of Neuroscience, University of Florida College of Medicine, Gainesville, FL 32610 USA; 4 McKnight Brain Institute, University of Florida, Gainesville, FL 32610 USA; 5 Normal Fixel Institute of Neurological Diseases, Gainesville, FL 32608 USA; 6 Duke Center for Neurodegeneration and Neurotherapeutic Research, Department of Pharmacology and Cancer Biology, Durham, NC 27710 USA; 7 Duke Institute for Brain Sciences, Duke University, Durham, NC 27710 USA; 8 Department of Medicine, Duke University and Department of Veterans Affairs Health Care System, Durham, NC 27710 USA

**Keywords:** Parkinson’s disease, Dementia with Lewy bodies, Parkinson’s disease dementia, Gastrointestinal tract, Immunity, Inflammation, Microbiome

## Abstract

Gastrointestinal (GI) involvement in Lewy body diseases (LBDs) has been observed since the initial descriptions of patients by James Parkinson. Recent experimental and human observational studies raise the possibility that pathogenic alpha-synuclein (⍺-syn) might develop in the GI tract and subsequently spread to susceptible brain regions. The cellular and mechanistic origins of ⍺-syn propagation in disease are under intense investigation. Experimental LBD models have implicated important contributions from the intrinsic gut microbiome, the intestinal immune system, and environmental toxicants, acting as triggers and modifiers to GI pathologies. Here, we review the primary clinical observations that link GI dysfunctions to LBDs. We first provide an overview of GI anatomy and the cellular repertoire relevant for disease, with a focus on luminal-sensing cells of the intestinal epithelium including enteroendocrine cells that express ⍺-syn and make direct contact with nerves. We describe interactions within the GI tract with resident microbes and exogenous toxicants, and how these may directly contribute to ⍺-syn pathology along with related metabolic and immunological responses. Finally, critical knowledge gaps in the field are highlighted, focusing on pivotal questions that remain some 200 years after the first descriptions of GI tract dysfunction in LBDs. We predict that a better understanding of how pathophysiologies in the gut influence disease risk and progression will accelerate discoveries that will lead to a deeper overall mechanistic understanding of disease and potential therapeutic strategies targeting the gut-brain axis to delay, arrest, or prevent disease progression.

## Background

In 1817, James Parkinson described six patients with “shaking palsy” and noted that some experienced constipation [1]. In one case, treating constipation alleviated the movement symptoms, linking gastrointestinal (GI) dysfunction to this neurodegenerative disease, later named Parkinson’s disease (PD) by Charcot [2]. At the time, cathartics were used for various ailments, as “ill humors” from the colon were thought to be causative. Since then, modern studies have confirmed the link between constipation and PD. Constipation affects 20–89% of PD patients, with increased incidence as the disease progresses [3]. Symptoms often begin over 10 years before motor impairments [4], and are partly caused by delayed colonic transit time [5]. Objective colonic dysfunction is more common than symptomatic constipation in PD [6]. Gastroparesis, a well-recognized comorbidity of PD, impacts gastric emptying and is thought to be less common than constipation, while esophageal and small bowel motility defects cause swallowing issues and bloating [7]. Contributing factors likely include neuro-hormonal dysregulation, gut microbial dysbiosis, inflammation, medications, and lifestyle shifts [8, 9]. CNS dysfunction is implicated in constipation, with treatments like deep brain stimulation showing GI improvements in some patients [10, 11].

Lewy body diseases (LBDs), including PD, dementia with Lewy bodies (DLB), and Parkinson’s disease dementia (PDD), can overlap in GI symptoms (Table 1). DLB is diagnosed when dementia occurs before or with PD motor symptoms, while PDD refers to dementia in established PD [12]. Constipation may be more prevalent in DLB than in PD [13], with one study suggesting DLB shows earlier onset of pre-motor constipation [14]. However, many longitudinal studies lack GI symptom data. Racial differences in PD are also under-explored, with some studies suggesting higher rates in Latino/Hispanic Americans and lower rates in Asians [15, 16]. While anosmia may be more common in African Americans [17], the role of GI symptoms across different races in LBDs remains largely unknown. There are currently little data on GI differences by race, particularly regarding PD [18]. Clinical scales like the Movement Disorder Society-Unified Parkinson’s disease rating scale (MDS-UPDRS) only indirectly address GI symptoms, such as swallowing difficulties [19]. The Scales for Outcomes in Parkinson’s Disease - Autonomic Symptoms (SCOPA-AUT) assesses GI function and is being incorporated into some cohorts like the Parkinson’s Progression Markers Initiative (PPMI). The SCOPA-AUT is recommended for use in studies, especially for DLB and PDD, where gut dysfunctions are more poorly understood [7]. The Global Parkinson’s Genetics Program (GP2) provides an opportunity to better understand the severity of GI dysfunction in LBD patients [20], which can be disabling for the patients.

**Table 1:** Gastrointestinal dysfunction in Lewy body diseases

Condition	Prevalence	Underlying Mechanisms	References
**Parkinson’s disease**			
Constipation	20–89%	Impaired colonic motility due to vagal and ENS dysfunction	[3, 21]
Gastroparesis	45–90%	Impaired gastric motility and pyloric sphincter relaxation due to vagal and ENS dysfunction	[22–24]
Dysphagia	9–77%	Impaired esophageal motility	[25, 26]
Sialorrhea	75%	Dysphagia	[27, 28]
Bloating	54%	Small intestinal bacterial overgrowth (SIBO) due to reduced intestinal transit, and malabsorption and constipation	[29, 30]
**Dementia with Lewy Bodies (DLB)**			
Constipation	Insufficient data	Insufficient data but likely due to impaired ENS function	[13]
Gastroparesis	Insufficient data	Insufficient data	
Dysphagia	90%		[31]
**Parkinson’s disease dementia (PDD)**			
Constipation	Insufficient data to distinguish from PD	Impaired colonic motility due to vagal and ENS dysfunction	
Gastroparesis	Insufficient data to distinguish from PD	Impaired gastric motility and pyloric sphincter relaxation due to vagal and ENS dysfunction	
Dysphagia	Up to 100%	Impaired esophageal motility	[31]

With the prevalence and importance of gut dysfunction in LBDs in mind, this review highlights key studies on the pathobiological origins of gut dysfunction in LBDs, exploring how gut interventions might impact comorbidity progression in PD, DLB, and PDD, with a focus on what is known and what remains to be discovered.

## Main text

### GI tract innervation and Lewy pathologies in the gut

#### GI connectivity

**Fig. 1:**
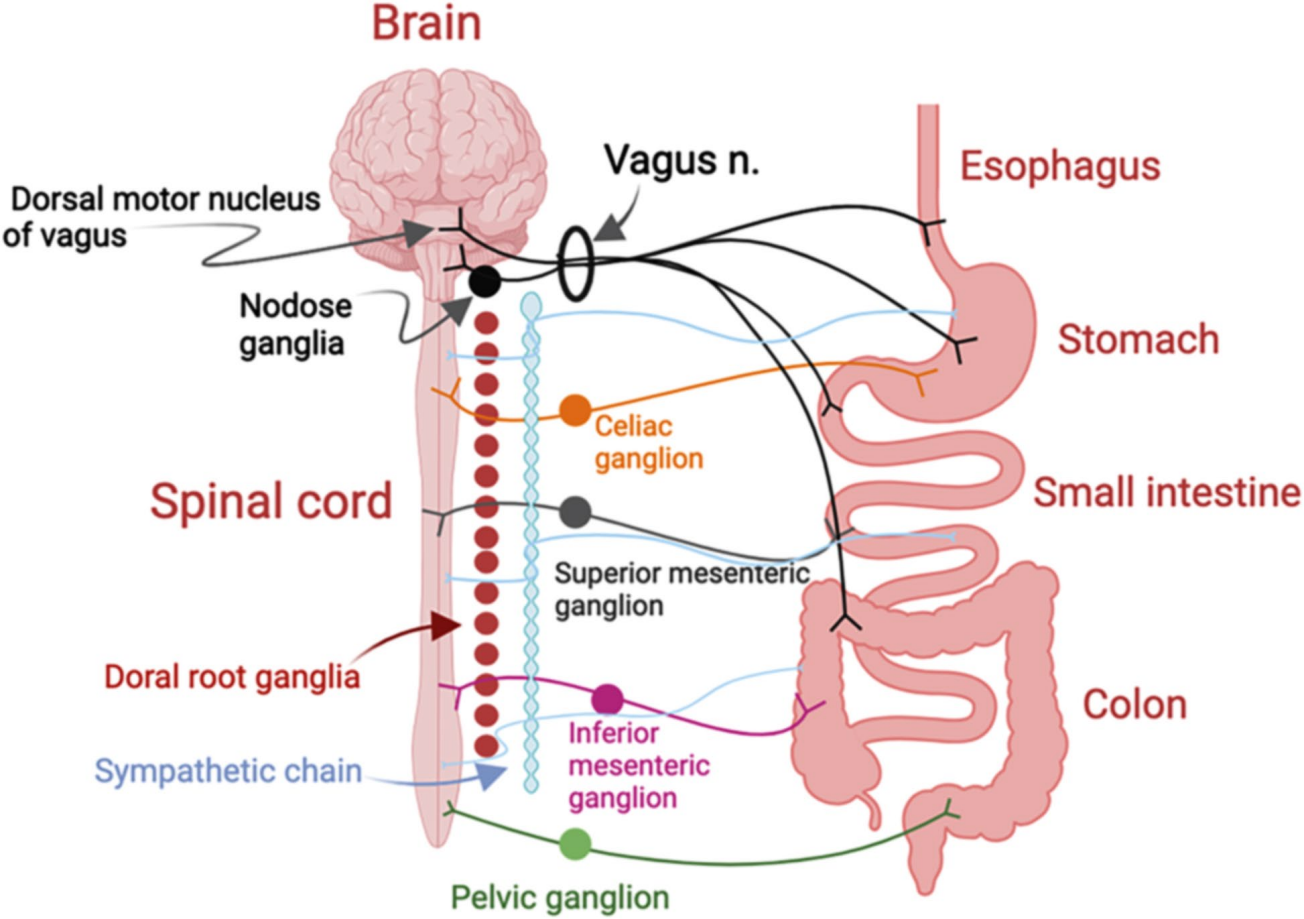
Graphical representation of the major neural pathways connecting the GI tract and brain. The vagus nerve contains both afferent and efferent fibers and innervates the esophagus, stomach, small intestine and proximal colon. Branched endings represent axonal endings. Cell bodies of the motor branch of the vagus reside in the dorsal motor nucleus of the vagus and those of the sensory branch are in the nodose ganglia. The celiac, superior and inferior mesenteric ganglia integrate sensory and motor inputs to the upper and lower GI tract and pathways carry signals to and from the distal colon and rectum. All nerves contain ⍺-syn and are subject to templating and propagating misfolded ⍺-syn thus representing routes of transport to susceptible brain regions. The vagus nerve richly innervates the upper GI tract and provides a direct route for ⍺-syn spread to the brain. (Modified from [33] and [34])

**Fig. 2:**
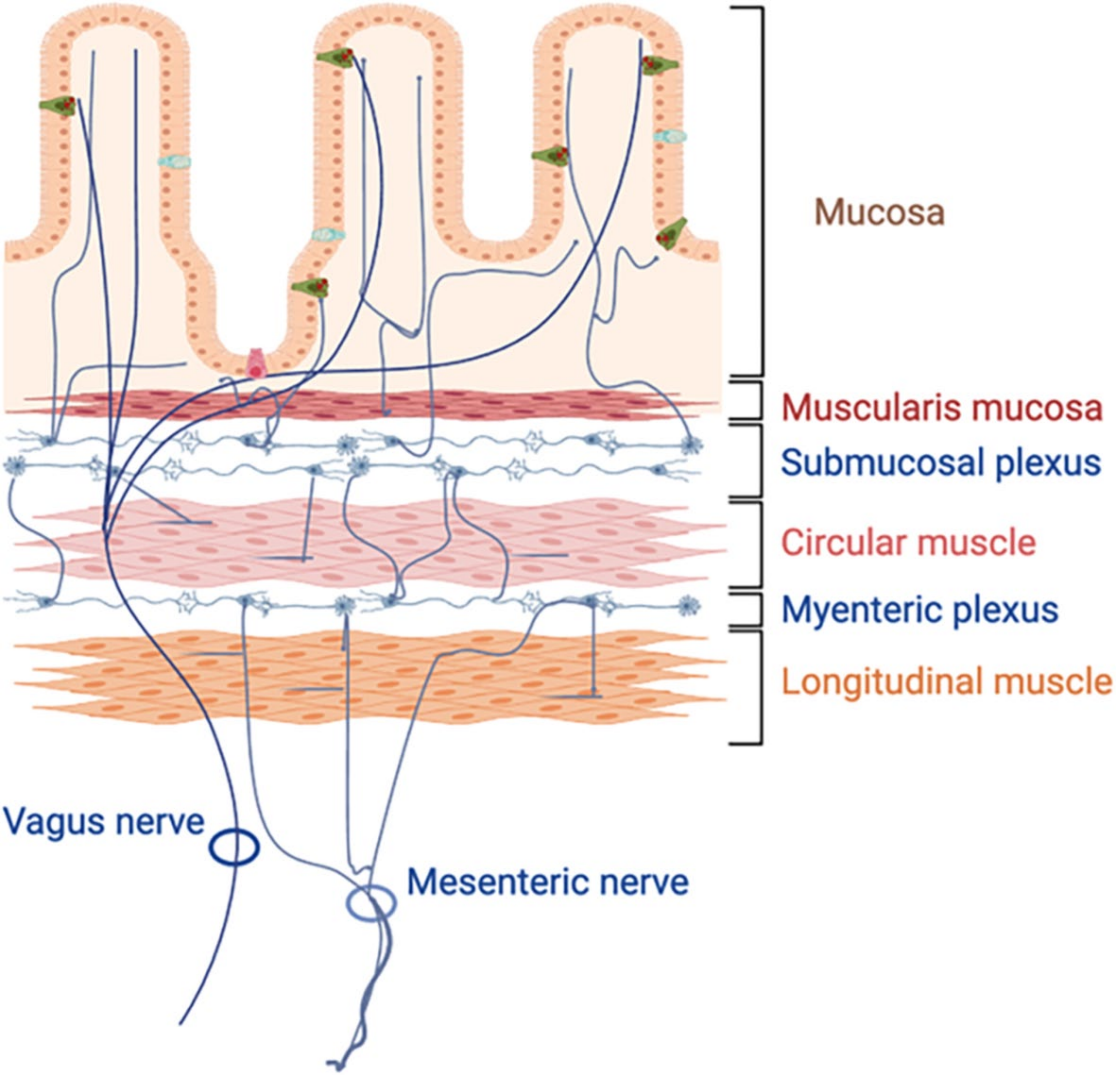
Diagram depicting the arrangement of the enteric nervous system (ENS) in the gut. In the small and large intestines, nerve cell bodies reside in ganglia of the myenteric or submucosal plexi. The myenteric plexus lies between the circular and longitudinal muscle layers of the bowel wall. Soma of the submucosal plexus are found in ganglia between the muscularis mucosa and circular muscle layer and extend neurites to crypts and villi of the bowel wall. Enteric and vagal fibers abut but do not penetrate the intestinal mucosa. In this location, any misfolded ⍺-syn (red dots) of mucosal origin (e.g., EECs, green) may spread to the nervous system. (Modified from [35])

The human gut communicates with the brain in a bidirectional manner through an integrated neural network connecting the ENS and the central nervous system (CNS) to relay signals and control digestive functions (Fig. 1). The ENS contains an estimated 200–600 million neurons, most of which are arranged in two major plexi [32] (Fig. 2). The myenteric plexus extends from the upper esophagus to the anus and forms a continuous neural network that controls gut motility. The submucosal plexus is comprised of a series of ganglia and connecting fibers in the small and large intestine but not the esophagus or stomach. These neurons regulate intestinal secretion and blood flow. Neurons from the ENS also project to prevertebral ganglia. Neural pathways connecting the gut to the brain fall into three categories: vagal, spinal thoracolumbar, and spinal lumbosacral, each of which carries afferent (sensory) and efferent (motor) signals [33] (Fig. 1).

The human abdominal vagus contains up to 50,000 axons which are a mixture of afferent and efferent neurons. The proximal gut is densely innervated by the vagus nerve in contrast to the distal small intestine and proximal colon which are sparsely innervated. There is little, if any, vagal innervation of the distal colon and rectum. Instead, colonic and rectal innervation is supported by spinal, sympathetic and parasympathetic nerves. Most vagal efferent fibers synapse with neurons in enteric ganglia although some directly innervate muscle cells. Key motor functions of the vagus include esophageal propulsion, lower esophageal and pyloric relaxation, gastric accommodation, and antral contractions, as well as stimulation of gastric and pancreatic secretion.

#### Lewy pathology in the gut

Lewy pathology was first discovered in the enteric nervous system (ENS) of the gastrointestinal tract in 1984 [36]. Several studies report abundant Lewy pathology (primarily based on pS129-⍺-syn staining) in enteric nerves throughout the GI tract, including submandibular glands, esophagus, stomach, small intestine, colon, and rectum of PD patients [37–40]. Pathology also occurs in the submucosa and myenteric plexi throughout the GI tract [41, 42]. Lewy neurites are found in vasoactive intestinal peptide (VIP)-expressing neurons [43]. Whereas dopaminergic neurons in the myenteric plexus might decrease in PD, VIP-containing neurons appear unaffected [44, 45], suggesting that gut dopaminergic neurons may be more susceptible to ⍺-syn-induced neurodegeneration as they might be in the central nervous system.

The exact prevalence of ⍺-syn fibrils in the GI tract broadly across LBDs is unknown [46]. Heterogeneity is certain to exist in these diseases, where tissue biopsy detection has found Lewy pathology in 75% of submandibular glands and only 6.7% in minor salivary glands [47]. Lewy neurites have been reported in the fundus, antrum, and duodenum [39], with ~ 60% of gastric biopsies from PD patients positive for phosphorylated ⍺-syn [48]. In colon biopsies, Lewy neurites were found in 4 of 5 PD patients, but absent in controls or those with constipation [38]. Phosphorylated ⍺-syn was detected in the colonic submucosal plexus in 21 of 29 PD patients, correlating with disease severity and constipation [49]. Lewy pathology was noted rostrocaudally in the colon, with 65% of patients showing Lewy neurites in the ascending colon, 42% in the descending colon, and 23% in the rectum [50]. Lewy bodies and neurites have been found 2–8 years before PD motor symptoms, suggesting that gut Lewy pathology may serve as a biomarker [46, 51]. While non-neuronal tissue like gut mucosal cells do not typically show Lewy pathology, rare ⍺-syn pathology was found in the colonic mucosa of three of nine PD patients, although the specific cell type was not identified [50]. Few studies have yet incorporated gut pathology analysis in DLB and PDD. This area is crucial for understanding gut dysfunctions in LBDs and how gut pathology intersects with broader pathological staging and timing. Additionally, the GI tissue used in those studies mentioned here is very small compared to the intestinal surface, meaning ⍺-syn deposits may be uneven, with some observations potentially underpowered to make broader conclusions (e.g., the lack of gut pathology in a case).

Besides histopathological analyses, positron emission tomography (PET) imaging has shown a loss of parasympathetic neurons in the small intestine and colon of PD patients [52, 53]. New assays for detecting misfolded ⍺-syn, such as seed amplification assays (SAA), real-time quaking-induced conversion (RT-QuIC), or protein misfolding cyclic amplification (PMCA) [54], hold promise for clinical PD detection in cerebrospinal fluid and brain [55, 56], but they are not widely used yet for human gut tissue [57]. These assays face the issue of pathological heterogeneity along the GI tract. Nevertheless, more sensitive assays to measure subtle ⍺-syn accumulation and/or aggregation may help detect early changes not visible through immunohistochemistry. Emerging technologies, such as optimized gut-tissue ⍺-syn seeding assays, PET tracers for misfolded ⍺-syn, and more sensitive measures of GI motor function, may offer crucial insights, especially early in disease.

#### Clinical observations supporting gut-to-brain pathology in LBDs

In addition to the indicated GI symptoms accompanying many cases of LBDs early in disease, clinical observations suggest that vagal transmission may be involved in disease pathogenesis. Using a large national medical registry, patients having undergone truncal vagotomy had a lower risk of developing PD than control subjects [58, 59]. However, highly selective vagotomy, in which only gastric branches of the vagus nerve were interrupted, leaving innervation of the intestine intact, did not affect PD risk, suggesting that a pathogenic factor possibly traveling via the vagus nerve originated distal to the stomach. The appendix harbors high ⍺-syn expression [60], and although it was reported that appendectomy might reduce the risk of PD [58], interpretation of these findings is not straightforward [61]. It is possible that removing the appendix eliminates a possible source for ⍺-syn pathology, however, it is not clear how appendicitis itself (which precipitates the need for appendectomy) might influence PD risk and progression. Independently, a recent retrospective cohort study reported that upper GI mucosal damage was associated with an increased risk of PD [62]. Endoscopic evaluation of 9,350 patients indicated that erosions, esophagitis, ulcer, or peptic injury of the esophagus, stomach or duodenum had a 76% greater risk of developing PD. The authors also found that subjects with mucosal damage were also more likely to have a history of proton-pump inhibitor and chronic nonsteroidal anti-inflammatory drug use, gastroesophageal reflux disease (GERD), constipation, smoking, dysphagia, and *Helicobacter pylori* infection, which each may contribute to PD risk. In complement, epidemiological studies report a higher prevalence of *H. pylori* infection in patients with PD [63, 64]. The importance of co-occurrent disruption of the epithelial barrier and inflammatory response and LBD risk may be supported through associations between inflammatory bowel diseases (including Crohn’s disease and ulcerative colitis) and PD [65]. Overall, these studies implicate bowel inflammation, of microbial or environmental origin, in LBD risk.

#### Origins of Lewy pathology in the gut

**Fig. 3:**
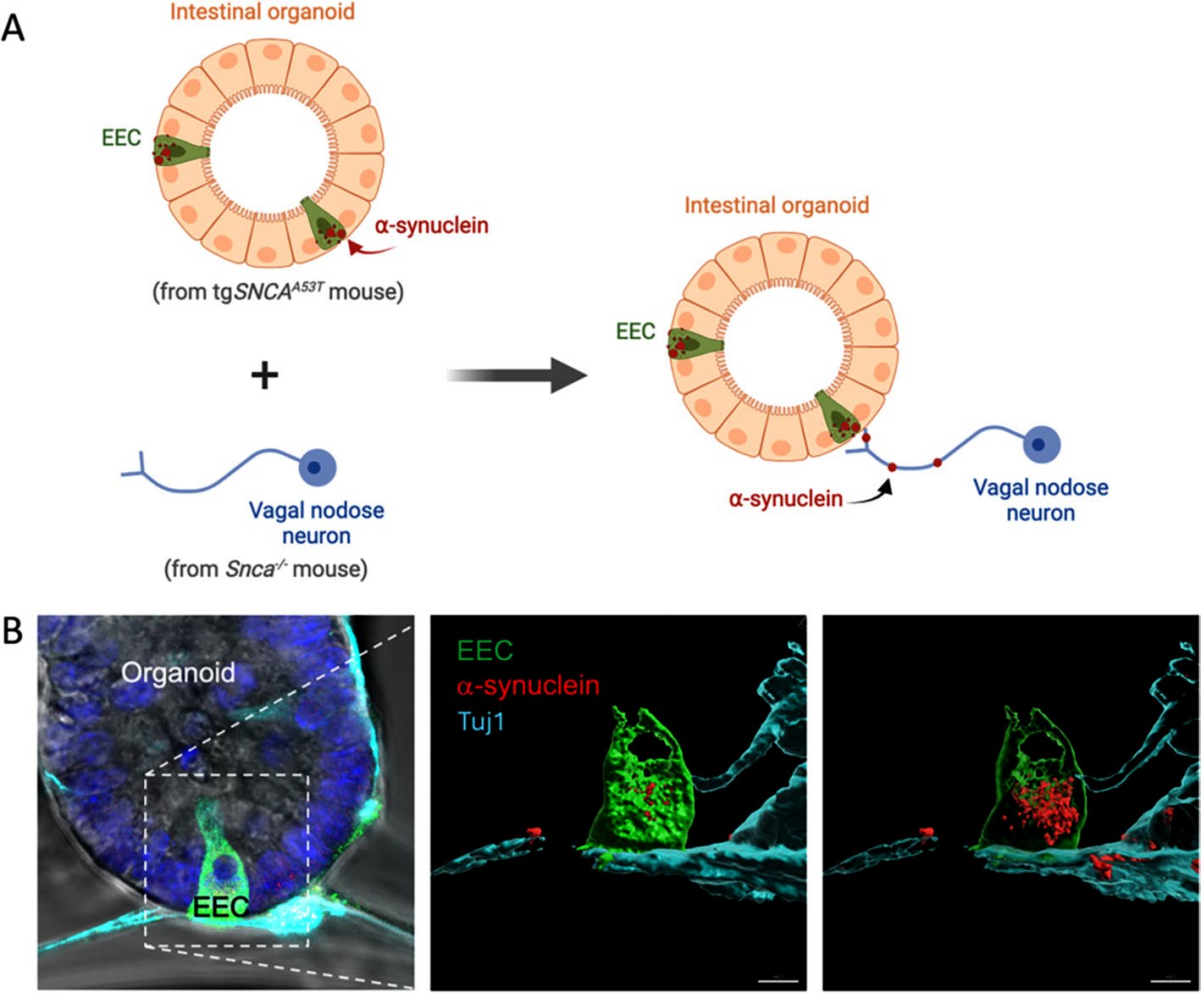
Co-culture of intestinal organoids and sensory neurons. **A** Cartoon depicting an experiment in which intestinal organoids from a transgenic mouse expressing human ⍺-syn (tg*SNCA*^*A53T*^) are co-cultured with nodose neurons from mice devoid of ⍺-syn (*Snca*^*−/−*^). Enteroendocrine cells (green) containing ⍺-syn (red) are dispersed among other cell types which are mostly enterocytes. Grown from intestinal crypt fragments or isolated stem cells, organoids form spheroids comprised of a single layer of mucosa surrounding a central lumen. **B** Nodose ganglion neurons (cyan) grown in gut organoid co-culture often contact EECs (green). Spread of ⍺-syn from tg*SNCA*^*A53T*^ EECs to nodose neurons from *Snca*^*−/−*^ mice has been demonstrated in culture [75]. This transer is depicted in the representative confocal images (scale bar is 3 μm)

Several possible scenarios may explain the unresolved phenomenon that GI dysfunction often precedes other symptoms across LBDs. The Braak hypothesis proposes (1) that an insult would first induce pathology in the olfactory bulb that could then spread into the brain [66], e.g., in “brain-first PD” [67, 68]. Alternatively, an insult would first induce pathology in a peripheral organ, like the gut, that could then spread to the brain [69], e.g., in “body-first PD” [67, 68, 70, 71]. This binary stratification may be incomplete as two simultaneous insults at sites from which pathology could spread bidirectionally might occur in some cases. DLB manifests with earlier pathology in limbic and/or neocortical areas early in disease, often with co-pathologies, further complicating a binary stratification (e.g., brain vs. body first) for LBDs. One possibility that cannot be ruled out is that ⍺-syn pathology emerges simultaneously across the body but progresses at different rates depending on the vulnerability of the tissue type and cells affected. As detection methods for pathological ⍺-syn continue to improve, for example in seeded aggregation assays, this scenario might be explored further in future studies. Alternatively, Lewy pathology initiating in the brain may descend to the gut, with gut cells intrinsically more vulnerable to pathology than other cells in the central nervous system in causing GI symptoms before motor disturbances. For example, dopamine signaling may be compensated in surviving neurons of the substantia nigra pars compacta [72, 73], despite early severe lesioning and synapse loss [74]. In contrast, compensation and normal function may be harder to maintain in the gut. A third scenario, hypothesized first based on pathological staging observations primarily with phosphorylated ⍺-syn (pS129-⍺-syn) staining, suggests pathology can occur first in the gut before spreading to the brain [37, 42, 51, 66]. In testing this third scenario experimentally, our laboratories developed methods to co-culture gut organoids expressing ⍺-syn together with nodose ganglia neurons lacking ⍺-syn expression (Fig. 3). For over a week, stable gut-to-neuron connections were observed between neurons and gut mucosal cells. In the model, the secretory gut cells transferred human ⍺-syn to adjacent neurons [75]. In potential support of these observations, a novel transgenic mouse model expressing human ⍺-syn under the direction of Villin-Cre expression in gut mucosal cells also successfully transferred ⍺-synuclein to enteric nerves, with transfer to the brain mitigated through full truncal vagotomy [75]. With these models developed, mechanistic studies are possible to further explore gut-to-brain transmission of ⍺-syn protein.

#### Emerging mechanisms of gut-to-brain progression of ⍺-syn pathology

Braak’s initial observations for predictable staging and progression of PD pathology [66, 69] and the idea that pathological ⍺-syn might spread from one cell to the next led Olanow and Prusiner, among others, to propose that ⍺-syn follows a prion-like spread, where misfolded ⍺-syn templates new pathology in neighboring cells [76]. Pathological protein folding conformations capable of spreading within a cell and into neighboring cells often have β-sheet structures (e.g., oligomers or fibrils), unlike native ⍺-syn, which is dominated by ⍺-helices. However, assigning ⍺-syn as a true prion is complicated by factors such as the lack of known infectious capacity in LBD tissue, the absence of ⍺-syn pathology in some interconnected neurons, and incomplete correlation between pathology and clinical manifestations [77, 78]. Consequently, ⍺-syn is often described as “prion-like.” Injection of mouse recombinant pre-formed fibrils directly into rodents can result in spread of pathology from the injection site, causing dopaminergic cell death in the substantia nigra [79, 80]. However, limited fibril-induced pathologies were observed in non-human primates after targeted enteric injections or intranasal administrations, despite high fibril doses [81, 82]. As there are substantial structural and functional differences between mouse and human ⍺-syn fibrils, the interpretation of studies in primates from those in non-transgenic rodents becomes difficult [83]. Spread of pathology induced by mouse ⍺-syn fibrils requires endogenous ⍺-syn, as it was absent in *Snca*-null mice [84]. Although some inconsistencies exist in the literature regarding the spread of ⍺-syn from the gut to the midbrain in rodent models [85, 86], most studies agree that ⍺-syn can propagate through anatomically connected neurons, particularly in the context of pre-existing aberrant transgenic ⍺-syn expression. The dorsal motor nucleus, locus coeruleus, and substantia nigra are interconnected and thought to play major roles in the initial spread of ⍺-syn pathology [87]. However, some studies suggest injected fibrils diffuse rapidly across the brain along a gradient, with lower doses causing slower pathology in different types of neurons, which might be misinterpreted as inter-neuronal spread [86]. Better models of cell-to-cell transmission are sorely needed using conformations of ⍺-syn closer to that found in the human brain to better understand mechanisms of gut-to-brain transmission.

With these caveats in mind, the export of ⍺-syn from cell to cell, crucial for hypothesized gut-to-brain spread, may occur via several mechanisms. Tunneling nanotubes have been proposed as one route for ⍺-syn transfer [88], where cell contacts are not necessary for transfer to recipient cells [89]. The release of ⍺-syn into the extracellular space is enhanced when lysosomal or proteasomal degradation is inhibited [90, 91]. Exosomes containing ⍺-syn have been observed, suggesting paracrine or other routes of transport to distant targets [92, 93]. Misfolded proteins may also be internalized by phagocytic cells, which can transport them to distant sites. While this has been shown for Aβ aggregates [94], to our knowledge it has not been described for ⍺-syn. Nevertheless, it does raise the possibility that ⍺-syn might spread independently of neuronal connections. Bulk engulfment by mobile phagocytes may also explain the phenotypes in fibril-injection models [86], although further study is needed for clarity.

### Intrinsic GI factors in LBDs

#### The vagus nerve

It is proposed that one way in which ⍺-syn spreads from the gut to the brain is via the vagus nerve, coursing through the dorsal motor nucleus of the vagus (DMV) in the medulla oblongata and from the lower brainstem to the substantia nigra and other brain regions including the cerebral cortex in both PD and DLB. Lewy pathology and nerve loss has been found in the vagus nerve [95, 96] and DMV [97, 98] of PD patients and might occur in the vagus nerve before pathology in other parts of the brain, including the substantia nigra [66, 69, 99–101]. Although less attention has been paid to the sensory branch of the vagus nerve in pathogenesis, there is evidence that the nodose ganglia may also be affected by Lewy pathology in PD [102, 103]. Less is yet known in DLB, but if there indeed is spread from the gut via the vagus, nodose involvement would be consistent with anterograde transmission of ⍺-syn. Injection of aggregated ⍺-syn into the stomach or duodenum of rats spread to the DMV through the vagus nerve [104] but has also been observed in the nodose, as well as the spinal cord and cardiac nerves [105], suggesting both antero- and retrograde spread. Taken together, these data suggest that ⍺-syn proteins have the potential to spread from the gut to the brain via the vagus nerve.

#### Mucosal cells

As a digestive organ, the initial nutritive functions of the gut occur at the interface of the gut lumen. The lumen is where food meets the mucosal epithelium. The esophageal mucosa is comprised of squamous epithelium in contrast to the stomach which contains a mixture of specialized secretory cells that secrete gastric acid and pepsin which begin to breakdown food to component parts. Enzymatic digestion occurs primarily in the small intestine and nutrient absorption is facilitated by the extensive surface area provided by villi. Enteric nerves such as intrinsic primary afferent neurons or vagal neurons that project to the mucosa do not penetrate the epithelial lining and are not exposed to the intestinal lumen. Rather they lie beneath the mucosa. In this position, they receive signals from gut mucosal cells that respond to nutrients, microbes, environmental toxicants, or other ingestants in the gut lumen.

#### Enteroendocrine cells

The best studied epithelial sensors of the gut are enteroendocrine cells (EECs), which respond to chemical, mechanical, and bacterial stimuli, to release hormones and/or neurotransmitters from their basal surface. EECs were originally classified by the hormones they produced [106]. In the stomach, EECs include gastrin-producing G cells and somatostatin-producing D cells. In the small intestine, the largest population are serotonin-producing enterochromaffin cells, but others produce cholecystokinin (CCK), secretin, and glucose-dependent insulinotropic peptide (GIP). Serotonin cells are also abundant in the colon, along with peptide YY- and glucagon-like peptide-producing L cells. EECs are flask-shaped or elongated, with an apical surface open to the lumen and a basal surface abutting the submucosa, which contains blood vessels, nerves, glia, immune cells, and fibroblasts. Their apical surface is covered with microvilli to sample luminal contents. The basal part of the cell contains vesicles, including hormone-containing dense core vesicles and small neurotransmitter granules. Like enterocytes, EECs arise from stem cells in the crypt and migrate up the villus over 3–5 days, undergoing apoptosis at the villus tip.

We discovered that EECs possess axon-like basal processes called neuropods that contain neurofilaments, mitochondria, secretory vesicles, and synaptic proteins [107]. Some EECs called ‘neuropod cells’ are synaptically connected to enteric neurons, including the vagus nerve [108]. In co-culture experiments, EECs and nodose neurons spontaneously formed physical connections that relayed electrical signals within milliseconds, indicating synaptic connection [109]. Synaptic connectivity was confirmed by rabies viral tracing, where rabies instilled into the gut lumen was taken up by EECs and transferred monosynaptically to the vagus nerve [109].

EECs express sensory receptors for small molecules on their surface. Located in the mucosa, they interact directly with intestinal microbes and environmental molecules such as dietary metabolites and xenobiotics [85]. Through signaling to the CNS, they are able to detect and differentiate between numerous metabolites [110]. Receptors for microbial metabolites like free fatty acids (FFARs) for short-chain fatty acids (SCFAs) and toll-like receptors (TLRs) for microbial components are found on EECs, allowing direct interactions with microbes [111, 112]. Some EECs also take up small dextrin antigens in vivo, suggesting they may sample the intestinal environment, including microbial products, toxicants, or other particles [113].

Although most mucosal cells are short-lived, EECs may last 60 days or longer [108]. The life cycle of EECs in humans can only be extrapolated from rodent models. However, EECs in rodents and humans express ⍺-syn protein [114], raising the possibility they could develop misfolded ⍺-syn, although a relatively short life would ostensibly preclude the formation of long-duration mature ⍺-syn inclusions. If misfolding occurs in EECs, pathogenic ⍺-syn could spread to nearby synaptic-like connections with enteric ganglia and vagal nerve endings [75]. Given that ⍺-syn regulation and misfolding are better understood in neurons, factors governing ⍺-syn expression and metabolism in EECs are almost entirely unknown. The normal function of ⍺-syn in EECs is not yet known, and gut phenotypes in mice lacking or overexpressing ⍺-syn in gut cells have not been fully evaluated. However, studies of ⍺-syn in EECs could help elucidate its role in PD. The primary function of ⍺-syn in neurons involves vesicle interactions and membrane binding, processes that may also be important in EECs.

### Extrinsic GI factors in LBDs

#### The microbiome as an integral component of the GI tract

The GI tract is deeply intertwined with the microbiome, a complex ecosystem of bacteria, archaea, viruses, and fungi. The bacterial portion alone consists of around 3,500 unique species in the human gut, with each person hosting roughly 100–300 unique species. These microorganisms are found in abundances that rival human cells in the body [115, 116]. The gut microbiome is estimated to encode 100-fold more genes than the human genome [116]. This vast genetic diversity enables the microbiome to influence nearly every organ system in the body. It plays a critical role in metabolic processes and immune development. For example, certain microbes are essential for developing immune cell subtypes in the intestines, periphery, and CNS, including microglia [117, 118]. The microbiome also influences the host production and metabolism of key GI metabolites like serotonin, mucins, and bile acids [119–121]. Additionally, specific microbes themselves generate vital metabolites such as biotin, indoles, and short-chain fatty acids (SCFAs), many of which humans cannot produce independently [122]. The composition of the gut microbiome is dynamic, changing throughout life, and influenced by diet, environment, genetics, and aging [123].

#### The gut metagenome in LBDs

Neurological diseases, particularly PD, are increasingly linked to certain bacterial communities, which may have distinct physiological effects contributing to disease development. In the last ten years, many independent studies have investigated the gut microbiome composition in individuals diagnosed with PD [124–129]. More limited datasets exist for LBDs other than PD [130, 131]. Stool and intestinal mucosal microbial communities have been assessed using 16 S rRNA profiling for bacterial populations or shotgun metagenomic sequencing for all available DNA, including bacteria, archaea, fungi, and some viruses. Nearly all studies comparing PD and controls show compositional differences in bacterial species. Several meta-analyses across independent studies have shown that the PD-associated microbiome is generally characterized by increased *Akkermansia* sp., *Bifidobacterium* sp., Enterobacteriaceae, and Lactobacillus sp., and decreased *Faecalibacterium* sp., *Lachnospiraceae* sp., and *Roseburia* sp. In DLB-associated microbiomes, increased *Ruminococcus* sp. and *Prevotella* sp. and decreased *Bifidobacterium* sp. and *Collinsella* sp. have been observed, however, these studies are more limited in size. Although bacteria are most studied, a few studies have assessed fungal or viral members in the PD-associated community [132–134]. Bacteriophage composition is different in the PD microbiome likely due to their relationship with bacterial hosts [133, 135]. Fungal communities appear less impacted in PD; however, the reasons, and consequences, for these alterations have not been described [132, 134].

Central questions regarding the development of LBD-associated changes in the microbiome remain: When do these microbiome compositions arise during disease, especially in relation to gastrointestinal dysfunction? Are these taxa enriched or depleted before disease onset, or through its progression? The pre-diagnosis period for PD is long, making longitudinal sampling expensive and burdensome. One possible approach is identifying high-risk individuals with genetic predispositions (e.g., PD-linked variants in *SNCA*, *GBA1*, and *LRRK2*), and tracking microbiome compositions as they age until PD symptoms emerge. While results would be limited to genetic forms of PD, they may reveal when the microbiome becomes impacted. Individuals at higher risk for LBDs affected by inflammatory bowel disease (IBD), hyposmia, constipation, and REM Behavioral Disorder (RBD) might be considered for longitudinal tracking of microbiome compositions [67, 70, 71]. Indeed, a few PD-associated bacterial species identified in individuals with RBD were different from controls [136, 137]. A recent analysis revealed shared depletion of butyrate-producing bacteria in PD and IBD microbiomes [138]. Another identified depleted SCFA-producing taxa in both individuals with constipation and PD [137]. As these bacteria have anti-inflammatory effects, their depletion may increase PD risk through inflammatory modulation. Together, these findings highlight the need for longitudinal microbiome studies in high-risk individuals to identify species that may impact disease risk and progression. While few longitudinal studies monitor the microbiome in pre-motor stages of PD, some have followed individuals through disease progression [139–141]. These studies show that microbiome structures persist over time, suggesting that compositional differences arise early in PD.

Another important question is what drives the initial shift in microbiome composition. Peripheral neuronal dysfunction due to ⍺-syn pathology could lead to slowed motility or barrier dysregulation, and alter the intestinal environment. Increased pro-inflammatory signaling might also affect the microbial community by releasing antimicrobials or metabolites. Medications for PD symptoms, as well as changes in diet due to physical difficulties or GI discomfort, could further impact the microbiome [142]. In addition, environmental exposures may act directly on native microbes, selecting for taxa, and instigating a long-lasting shift in the community structure. Longitudinal studies of at-risk populations are needed to clarify the microbiome’s role before PD onset.

A key question remaining from all these studies is whether these differences contribute to PD risk and/or progression. Metagenomic sequencing has shown that not only is the microbiome composition altered in PD, but its genetic capacity is also changed, with increased proteolytic pathways and genes stimulating pro-inflammatory immune responses [124–127, 135, 143–145]. Understanding the dynamics of these microbiome features throughout disease stages will enable new hypotheses about the microbiome’s contribution to etiology and/or progression.

#### Microbiome manipulations in LBD mouse models

To test whether the microbiome has pathological consequences to ⍺-syn pathology and associated neurodegeneration, gnotobiotic mouse models have provided a useful platform for precise manipulation of the microbiome. Studies using these, and other microbiome manipulations, have begun to yield foundational evidence to support the hypothesis of a microbiome contribution to LBD-risk and progression. For instance, germ-free derivation or broad-spectrum antibiotic treatment have been shown to limit ⍺-syn pathology and neurodegeneration across both genetic and toxicant-induced models [146–148]. In both genetic and toxicant-induced models, microbiomes derived from individuals with PD can exacerbate neurodegeneration and motor impairments, compared to animals exposed to microbiomes derived from persons without disease [146, 149]. Indeed, exposures to specific microbes, such as those from the *Enterobacteriaceae* family, can exacerbate ⍺-syn pathology across a number of model systems (worms, mice, rats), in both ⍺-syn based models and toxicant exposures [150–152], supporting the concept of pathogenic organisms which may trigger ⍺-syn misfolding. Recent studies of uropathogenic *E. coli* further demonstrated an experimental link between infection and ⍺-syn pathology within the innervation of the genitourinary tract, at least in the context of the synucleinopathy, multiple system atrophy [153].

#### Gut bacterial amyloids

Many bacteria produce amyloidogenic proteins crucial for bacterial physiology, including surface attachment and biofilm formation [154]. Some amyloid-producing bacteria have been found to be enriched in the microbiome of individuals with PD [125, 155]. Given evidence for cross-seeding by various amyloidogenic proteins, where β-sheet conformers of one protein can promote the formation of β-sheet conformers of ⍺-syn, bacterial amyloids are hypothesized to interact with ⍺-syn in aggregation pathways [154, 156]. Recent evidence from a genetic screen for *E. coli* mutants unable to trigger ⍺-syn aggregation in *C. elegans* identified genes for the curli amyloid as necessary for this process [150]. This is replicated across model systems, including rats and mice, and is exacerbated with a low-fiber diet [151, 152, 157]. Biochemical evidence suggests a transient, direct interaction between monomeric curli proteins and ⍺-syn [158]. Recently, diverse gut microbial amyloids were shown to increase ⍺-syn accumulation in *C. elegans* [159], highlighting that bacterial amyloids beyond curli may also have pathogenic potential. Microbial amyloids are known to interact with pattern receptors on immune cells, including macrophages and monocytes, potentially contributing to the gut’s inflammatory environment. This is supported by evidence showing that LPS (a major component of Gram-negative bacteria) can interact with ⍺-syn and promote its aggregation [160], and that inhibiting microbial receptors such as TLR2 and TLR4 can protect against pathology in animal models [161, 162]. Further studies are needed to clarify the contributions of immune interactions and direct ⍺-syn aggregation by microbes or their surface molecules.

#### Microbial-metabolite and toxicant interactions

The microbiome is responsible for production and metabolism of a plethora of metabolites with activities locally, systemically, and in the CNS [122]. One well described class of metabolites, the SCFAs including propionate and butyrate, have actions on many physiologies. Through actions on FFARs and histone deacetylases (HDACs), SCFA-derived signaling promotes immune development and signaling, for instance, the production of regulatory IL-10 and the development of regulatory T cells. SCFA signaling is necessary for the homeostatic development and functionality of microglia in infection and amyloid models [117, 163]. SCFAs are derived from the microbial-dependent fermentation of dietary fiber, including starches such as wheat bran, inulin and potato starch, as well as fructo-oligosaccharides. The PD-associated and DLB-associated microbiomes are observed to be depleted in those bacterial taxa responsible to produce these molecules, leading to the hypothesis that loss of SCFA production may result in a predisposition to neuroinflammation and contribute to LBD pathology. Persons with PD have depleted concentrations of SCFA in stool [129, 164, 165]. High-fiber dietary interventions provide benefits to enrich depleted taxa in people living with PD and improve GI motility and markers of inflammation [166]. Further, in an ⍺-syn-overexpression mouse model, high-fiber diet limits ⍺-syn accumulation and motor deficits [167]. Given that dietary fiber interventions are relatively easily administered, these may represent an avenue to beneficially modify GI and related immune responses that might contribute to LBD risk and progression.

#### Toxicants (including microplastics)

Environmental exposures to toxicants, such as pesticides and herbicides, represent a significant risk for LBDs [168]. An estimated 10–20% of PD incidence in southeast US and South American populations is attributed to pesticide/herbicide exposure [169, 170]. The microbiome sits at the interface between the external environment and the body, ready to interact directly with PD-linked toxicants. Microbial metabolism can convert xenobiotics into inactive or hyperactive forms [171]. In PD, it is unknown how the gut microbiome metabolizes PD-linked toxicants like 1-methyl-4-phenyl-1,2,3,6-tetrahydropyridine (MPTP), rotenone, trichloroethylene (TCE), or paraquat. However, in animal models, antibiotic treatment or germ-free status limits pathology in some toxicant-triggered nigral degeneration models [148, 172–175], suggesting the microbiome mediates or limits the capacity to modulate toxicological outcomes. Experimental work is needed to identify whether certain microbiome compositions alter sensitivity to these exposures and the associated inflammation and neurotoxicity, and whether this is due to direct metabolism by microbes or through signaling to the host. One possibility is exploring whether pre-existing microbiomes predict toxicant exposure effects on PD risk. Toxicant exposures themselves shift the microbiome composition [176], with alterations similar to those in PD, such as increased *Lactobacillus* and *Bifidobacteria* and decreased SCFA producers [177, 178].

As public awareness of pesticide hazards grows, microplastic pollution brings new focus to emerging environmental risk factors for PD progression [179]. Microplastics and nanoplastics from plastic waste are increasing across the environment [180]. These small particles can harbor and transport harmful chemicals, including “forever” toxicants like phthalates and per- and polyfluoroalkyl substances (PFAS), so named for their slow rate of environmental decomposition. Recent studies show that plastic nanoparticles can recruit ⍺-syn protein into β-sheet-folded fibrils associated with toxic ⍺-syn oligomers, potentially contributing to disease spread [181]. Research on plastic pollution’s role in PD is nascent, but the GI tract may be heavily impacted by microplastics, especially those microplastics with low bioavailability and nanoplastics internalized in cells like EECs. Initial studies suggest microplastic exposure alters the mouse gut microbiome, eliciting pro-inflammatory cascades that negatively affect the brain and microglia [182]. As microplastic and nanoplastic concentrations rise together in our environment with linked toxicants, these early studies suggest a need for further exploration into whether microplastics and their additives can trigger a PD-associated microbiome composition, inducing inflammation, and directly contributing to ⍺-syn aggregation in the gut.

### GI inflammation in LBDs

#### Gut macrophages and infiltrating blood monocytes

The ENS contains specialized macrophages that maintain homeostasis and respond to infections [183–185]. When gut homeostasis is disrupted, blood monocytes migrate to the inflamed gut, becoming pathogen-defense-associated macrophages [186]. Individuals with Crohn’s disease (CD) have fewer blood monocytes but more intestinal macrophages [187], indicating monocyte migration during inflammation. Monocytosis and monocyte infiltration often signal GI inflammation and impact disease outcomes [188]. While blood-derived monocytes’ role in GI dysfunction is still uncertain, further research in animal models and human studies is necessary. Gut macrophages have been implicated in PD progression [129, 164, 189]. ENS macrophages may spread enteric ⍺-syn aggregates to the CNS [190]. CNS-resident microglia perform synaptic pruning and activate immune responses that contribute to neuronal death [191–193]. While microglia have been linked to both neuroprotective and harmful functions in neurodegenerative diseases [194, 195], the same is not clear for ENS macrophages. Few studies have examined ENS macrophage responses to ⍺-syn dysfunction and its role in PD-like degeneration. Early research in mice using pre-formed ⍺-syn fibrils injected into the gut did not report macrophage involvement in modulating this process [196]. These cells likely played a role in clearing debris, antigen presentation, and modulating gut inflammation. A recent study showed that ⍺-syn fibrils triggered enteric pathology and disrupted ENS neuron-macrophage interactions, leading to macrophage upregulation of synaptic pruning via C1q [197]. However, macrophage exhaustion due to ⍺-syn overexpression in the gut resulted in pathology spread and intestinal dysmotility. Further research is needed to understand how ⍺-syn disrupts clearance mechanisms and blocks anti-inflammatory responses that limit PD and/or LBD risk.

#### Adaptive immune cells and autoimmune mechanisms

The role of adaptive immune cells, B and T cells, in triggering or promoting ⍺-syn pathology is less well understood compared to the function of innate immune cells. In animal models of autoimmune neurological conditions, T cells home to the intestines and directly interact with other cells in the gut microenvironment prior to migrating into the CNS [198, 199]. Similarly, peripheral monocytes migrate into the CNS and transcriptionally transition into antigen-presenting cells within the brain parenchyma [200]. Although it has been reported that individuals with PD develop ⍺-syn-specific T cells detectable in the blood [201, 202], it is not known whether these T cells traveled to the gut first where they were presented with modified epitopes of ⍺-syn by gut macrophages or by other antigen-presenting cells in the blood or the brain. An in-depth understanding of the mechanisms involved in this central-peripheral neuroimmune crosstalk and its dysregulation as a function of aging and/or exposures will be needed for development of effective immunomodulatory therapies for the PD and LBD clinics and should be prioritized for testing in murine and other models of ⍺-syn-mediated dysfunction.

#### Molecular mimicry

Mimicry can occur when similarities between foreign and self-peptides activate autoreactive T or B cells in a susceptible individual [203]. This is a primary mechanism by which infections or chemicals can induce autoimmunity and may play a role in generating ⍺-syn reactive T cells [201, 202]. Other factors, such as host genetics, tolerance breakdown, and persistent gut infections, may also contribute to autoreactive T cells recognizing modified ⍺-syn peptides. Mucosal damage triggering a local gut inflammatory response may promote molecular mimicry, through affecting local ⍺-syn misfolding and expression, reducing clearance of misfolded forms, or other mechanisms. Mucosal damage may also cause more systemic effects via inflammatory mediators that influence the enteric or central nervous systems, broadly modifying PD and/or LBD risk. Given the complex gut microenvironment and its crosstalk with the CNS, these factors may act together to increase response variability across populations.

### Therapeutic implications

As the broader field of neurodegeneration acknowledges that disruption of proteostasis in the gut-brain axis poses a significant threat to successful brain aging, the next question is whether these processes can be modified to mitigate neurodegeneration risk and when interventions are most effective. With respect to the gut-brain axis in LBDs, studies suggest that diet regimens with high fiber, low sugar, omega-3 fatty acids, and polyphenols promote a healthy gut microbiome and reduce inflammation [204]. Dietary interventions, including the prebiotics lactulose, inulin, and oligosaccharides can promote disease resilience [205]. Although prebiotics in pre-clinical models have shown promise for modulating anxiety and stress [206], evidence linking them to neurodegeneration risk modification is limited. Similarly, fecal microbiota transplants (FMTs), used in treating *Clostridioides difficile* infections, have been studied for their effect on microbial diversity in inflammatory bowel disease (IBD) and multiple sclerosis (MS), but their effectiveness in LBDs is less clear [207]. Given immune involvement, targeting pattern recognition receptors and/or the downstream cytokine signaling, such as TNF, may also prove beneficial. Large, randomized studies in diverse populations are necessary to determine if these interventions can delay or prevent disease progression at early stages. Ongoing clinical trials targeting the gut-brain axis are discussed elsewhere [204].

## Conclusions

**Fig. 4:**
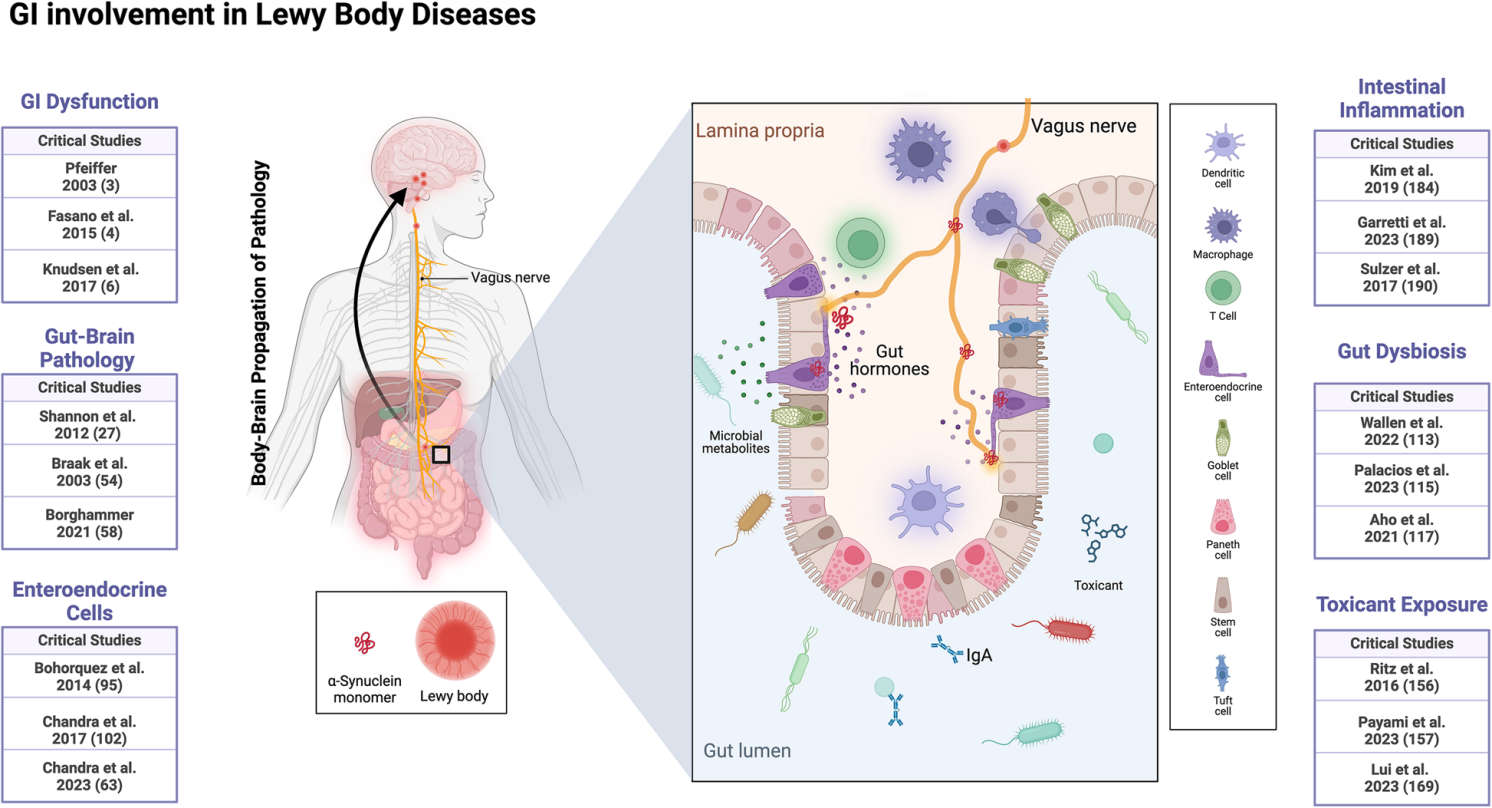
Publications supporting key concepts highlighted in this review for GI involvement in the pathogenesis and pathophysiology of Lewy body diseases

The earliest descriptions of LBDs noted clear gut involvement. Despite the known association between GI dysfunction and LBDs, many longitudinal studies still lack detailed information about GI pathologies in early PD and their predictive role in LBD progression. A mechanistic link between GI dysfunction in PD and DLB has begun to emerge (Fig. 4). PD pathology seems to follow a predictable staging pattern, with the spread of toxic factors like misfolded α-syn from the gut to peripheral nerves contributing in some cases. The hypothesis that PD pathology spreads in a prion-like manner from peripheral organs via the vagus nerve or other neurons is supported by the detection of ⍺-syn pathology in olfactory and enteric neurons, and the ability of misfolded ⍺-syn to transfer between the gut mucosa, and neurons in the enteric and central nervous system. EECs, which express ⍺-syn, may contribute to the initial pathobiological events by transferring cargo to adjacent nerves. Disruption of the gut microbiome is associated with PD and has potential to contribute to pathogenesis, but it is unknown if the same microbiome alterations are present at similar disease stages in PDD and DLB. Whether microbes or infections initiate ⍺-syn pathology is still uncertain. Inflammatory features, often accompanying dysregulation of the microbiome and GI diseases, influence PD progression and aggravate symptoms. Targeting specific inflammatory pathways at the gut-brain interface holds promise for therapies aimed at halting or blocking early-stage disease progression. Identifying emerging environmental exposures (such as pesticides, solvents, plastics, etc.) that increase LBD risks could help reduce future risks in specific populations. Current PD and LBD treatments focus on alleviating symptoms but do not slow disease progression. A better understanding of the steps involved in disease initiation, especially whether ⍺-syn is required for the pathogenic process, will likely accelerate the development of effective disease-modifying strategies, without hindering interventions targeting the gut-brain axis to delay disease progression.

## Data Availability

Not applicable.

## Abbreviations

GI: Gastrointestinal
⍺-syn: Alpha-synuclein
PD: Parkinson’s disease
LBD: Lewy body disease
DLB: Dementia with Lewy bodies
PDD: Parkinson’s disease dementia
MDS-UPDRS: Movement Disorder Society-Unified Parkinson’s disease rating scale
ENS: Enteric nervous system
GERD: Gastroesophageal reflux disease
MD: Mucosal damage
PET: Positron emission tomography
SAA: Seed amplification assay
RT-QuIC: Including real-time quaking-induced conversion
PMCA: Protein misfolding cyclic amplification
DMV: Dorsal motor nucleus of the vagus
MD: Mucosal damage
GERD: Gastroesophageal reflux disease
CNS: Central nervous system
ENS: Enteric nervous system
EECs: Enteroendocrine cells
SCOPA-AUT: Scales for Outcomes in Parkinson’s Disease- Autonomic Symptoms
PPMI: Parkinson’s Progression Markers Initiative
CCK: Cholecystokinin
GIP: Glucose-dependent insulinotropic peptide
FFARs: Free fatty acid receptors
SCFAs: Short-chain fatty acids
TLRs: Toll-like receptors
IBD: Inflammatory bowel disease
REM: Rapid eye movement
RBD: Rapid eye movement behavioral disorder
LPS: Lipopolysaccharide
HDACs: Histone deacetylases
PFAS: Per- and polyfluoroalkyl substances
TCE: Trichloroethylene
MPTP: 1-methyl-4-phenyl-1,2,3,6-tetrahydropyridine
